# Effective integration of sexual reproductive health and HIV prevention, treatment, and care services across sub-Saharan Africa: where is the evidence for program implementation?

**DOI:** 10.1186/s12978-019-0709-6

**Published:** 2019-05-29

**Authors:** Didier Mbayi Kangudie, Hugues Guidigbi, Sheila Mensah, Abdul A. Bala, Richard Delate

**Affiliations:** 1 United States Agency for International Development, West Africa Mission, Regional Health Office, Accra, Ghana; 2 United Nations Population Fund East and Southern Africa Regional Office, Sandton, South Africa

## Introduction

Ending AIDS as a public health threat while aiming for universal access to Sexual Reproductive Health (SRH) remains a dual strategic goal of both HIV/AIDS and SRH programming. Advancing this goal across the two programming areas will require deliberate interventions to connect implementation at policy, systems, funding, coordination, management, service delivery and monitoring levels.

In 2017, Sub-Saharan Africa was home to 25.6 million people living with HIV [4]. This represents 69.6% of the people living with HIV in the world. The level of new infections among children remains high with 159,000 new infections in 2017, despite the progress made during the past two decades to eliminate mother-to-child HIV transmission [4, 6, 7]. The global community survey carried out in 2014 on SRH priorities among women living with HIV to inform WHO guidelines, revealed 56.7% rate of unplanned pregnancy, with only 55.3% of women living with HIV having benefited from practical support for safe methods of conception. At three large maternity centers in the Eastern Cape, South Africa, the prevalence of unplanned pregnancy among women living with HIV was 71% (Oladele Adeniyi et al.) [8].

Additionally, sub-Saharan Africa has a low modern contraceptive prevalence rate estimated at 28% [2] and continued high unmet need for family planning (FP) at 22%, almost double the global average [3]. The journey to ending AIDS and ensuring universal access to SRH including FP by 2030 will require strategic investment choices and smart integration models.

Since the 2004 Glion Call to Action on the linkage between FP and Prevention of Mother-to-Child Transmission of HIV (PMTCT), several initiatives and policies have been put in place to facilitate SRH and HIV integration [5]. What constitutes service integration has long been a subject of debate. Johnson, Varallyay & Ametepi (2018) have tackled it with a literature review that revealed how the lexicon of terms referring to concepts related to “integration” is varied, and its definition is not uniformly understood. The related term most commonly used, sometimes interchangeably, is the concept of “linkages”. The World Health Organization made an attempt to distinguish between these two terms, defining “linkages” as a concept that encompasses more broadly the synergies that exist between sexual and reproductive health and HIV policies, programs, services, and advocacy efforts while defining “integration” as one level more specifically focused on targeted services and/or programs that can be joined together to ensure and maximize collective outcomes by offering more comprehensive services. Such definition requires specific organizational structures and management procedures to support such enhanced service delivery [1].

Despite insufficient data to fully evaluate the effect of SRH and Rights (SRHR)/HIV integration on unplanned pregnancies comparing outcomes from integrated to non-integrated sites, several studies demonstrated reduction in costs for service delivery, increased client knowledge, and increased use of modern contraceptive methods (Haberlen, Narasimhan, Beres & Kennedy, 2017) [23]. To realize higher levels of operational efficiencies, project implementers can learn from documented successful service integration models. As acknowledged by Johnson, Varallyay & Ametep (2018), policy recommendations from governments, donors, international organizations, and normative bodies explicitly support the integration of HIV and reproductive health service delivery, particularly family planning. There is recognition that integration is needed to support women‘s and men‘s reproductive health needs, to push the HIV epidemic back with the goal of ending AIDS as a public health threat and to reach universal access to SRH by 2030 as per the Sustainable Development Goals (SDG) [1].

A compilation of articles published in *Reproductive Health* and *BMC Public Health* examines the integration of SRH and HIV/AIDS from different perspectives. From the integration of family planning as a second prong of the prevention of vertical transmission of HIV including among female sex workers, to the concept of safer pregnancy in HIV-affected individuals, through the integration of SRH in the minimum package of HIV prevention, care and treatment. The articles also examine the design of innovative approaches for integration in community-based service delivery models. Defining success in service integration has been elusive and the experiences shared in these articles cut across several countries and geographic focuses including in Botswana, Cameroon, Kenya, Malawi, South Africa, Tanzania, Uganda and the United States. Additionally, one systematic review and one literature review explore respectively the specific issue of integration of HIV testing into family planning and the issue of integration of Adolescents and Young Women SRH in emergency settings.

## Discussion

Effective integration of HIV and SRH services requires not only behavioral change interventions for health care providers (Provider Behavioral Change) but also increased understanding of beneficiaries to respond adequately to their needs based on their knowledge, attitude, and risk perception. As demonstrated by González, Kadengye & Mayega, (2019) in their nationally representative household survey focused on young Ugandans, high levels of SRH/HIV knowledge and risk perception did not curb risky sexual behaviors despite the context of generalized HIV epidemic (prevalence of 2.1% among 15–24 years old). The authors asserted that effectiveness gaps in the integrated SRH/HIV response should be addressed holistically at individual and structural level [9].

A marginalized group among key populations in Sub-Saharan Africa, women who inject drugs, received attention in this supplement. Sylvia Ayon et al. (2019) used action research to identify the process, impacts, and challenges of integrating SRH into community-based HIV prevention and harm reduction programs in coastal towns in Kenya. Their findings highlight low utilization of family planning and other SRH services, and provide critical insight on the acceptability and opportunities for successful integration at community level [19].

A cross sectional study conducted in Kenya by Raymond Mutisya et al. assessed the level of integration of family planning across six other services delivery areas (ANC, maternity wards, postnatal care clinic, child welfare clinic, HIV counselling and testing, HIV/AIDS services in comprehensive care clinic settings). Their findings validate what has already been documented on the positive correlation between provider knowledge, skills and attitude and service quality [15].

A systematic review of the integration of HIV testing services into family planning services by Narasimhan et al. (2019) revealed that HIV counselling and testing was generally higher in integrated sites as compared to non-integrated sites, including in adjusted analyses through outcomes varied slightly across studies. Similar to Kiersten Johnson et al. findings, this review concludes that global progress and success for reaching SRH and HIV targets depends on progress in Sub-Saharan Africa where women bear a high burden of both unintended pregnancy and sexually transmitted infections, including HIV [16].

In a 2015 State of the World Report, UNFPA stated that the many crises, wars, and natural disasters around the globe, and especially in Africa, are leaving women and adolescent girls facing a significantly heightened risk of unwanted pregnancy, maternal death, gender-based violence, and HIV acquisition. It is only befitting that this supplement includes a literature review by Roxo, Walker, Mobula, Ficht & Yeiser, (2019) on prioritizing the SRH and rights of Adolescent Girls and Young Women (AGYW) within treatment and care services in emergency settings. Their review revealed that the plurality of competing needs in emergency settings crowds out dedicated time and space to effectively integrate HIV and SRH interventions and that greater political will is required to advance the integration agenda [20].

Two of the supplement’s articles discuss key findings on the availability of family planning and HIV-related integrated services in Sub-Saharan Africa, with one addressing the quality component. Studies by Kanyangarara, Sakyi & Laar, (2019) and Barden-O’Fallon, Mejia & Close, (2019) restricted analyses to health facilities offering HIV/AIDS care and support, not just HIV testing and counseling, and FP services. Using secondary analysis of Service Provision Assessments (SPA), Service Availability and Readiness Assessments (SARA) (2012–2015) for 10 countries including six in West and Central Africa), Kanyangara et al. found that 93% of facilities offering HIV care and support services also reported offering integrated services, but merely 29% were classified as having onsite integrated FP services based on the availability of structural and process of care inputs (e.g., equipment, guidelines, trained providers, and FP commodities). Further, 94% of facilities reported routinely offering FP counselling to HIV/AIDS clients and 80% had three or more contraceptive methods in stock at the time of the surveys [17, 22].

Matching SPA measures and a Quick Investigation of Quality indicators for FP quality in integrated compared with non-integrated lower-level HIV/AIDS care and support facilities in Malawi (2013–2014) and Tanzania (2014–2015), Barden-O’Fallon et al. found that 79% of facilities in Malawi and 38% in Tanzania offer FP services. In keeping with the Bruce/Jain Framework of Quality Care, 22 quality indicators were analyzed and showed integration status was significantly associated with three indicators for Malawi: “facility has all (approved) methods available: no stock outs”, “facility has received a supervisory visit in the past 6 months”, “look and write on client record”. For Tanzania the indicators associated with integration status were “facility has adequate storage of contraceptives and medicines”, “facility has all (approved) methods available: no stock outs”, “waiting time acceptable (negative)”, and “facility has mechanisms to make programmatic changes based on client feedback” [17].

Two of the articles reviewed the knowledge and use of safer conception methods (SCM) among HIV-infected individuals on how to accommodate their safer conception needs. Both studies by Gwokyalya et al. (2019) and Schwartz et al. (2019) highlighted the need for more education and making available different methods of SCM to people living with HIV. Of the 5198 number of women interviewed at the health facilities in Uganda, 74.1% had knowledge of SCM but the number decreases to 42% with those of knowledge of more than one method. The study also underscored the lack of the involvement of partners in serodiscordant relationships in the choice and intent to use SCM. They emphasized the lack of qualified staff to undertake some of the SCM such as sperm washing. The study highlighted the increasing challenges by individuals with HIV to fulfill their fertility desire and concluded that the knowledge and use of SCM among HIV+ women in care is low. Efforts to improve HIV status disclosure, integration of safer conception into FP and HIV services, and regional efforts to promote sensitization and access to safer conception can help to increase uptake of safer conception methods [18, 21].

With its four-pronged approach, PMTCT prevents 90% of new HIV infections among children and thus contributes to the AIDS-Free Generation. PMTCT is also at the crossroad of antenatal and postnatal care services, FP, and HIV prevention care and treatment services. Three manuscripts in this supplement address the issue of PMTCT through the lens of antenatal, HIV, and sexual and reproductive health services integration. The authors, Rwema et al. (2019), and Parmley et al. (2019), assessed the PMTCT cascade and investigated factors influencing antenatal care services seeking behavior in a context of high HIV prevalence among female sex workers (FSW) in Port Elizabeth, South Africa [10, 11].

Rwema et al.(2019) found that 61% of FSW were infected with HIV and 52% of them knew their HIV status before the study. A gap of 40% was found regarding the systematic use of condoms by non-HIV-infected FSW with their clients and a gap of 43% in the use of long-term modern contraceptive methods among FSW living with HIV. Parmley et al.(2019) found in a similar context in Port Elizabeth, South Africa, a late discovery of pregnancy (at 4 to 7 months) among FSW living with HIV and a 40% antiretroviral therapy coverage among them. The influencing factors identified were alcohol and psychoactive substance use, as well as dissatisfaction with past health care experiences [10, 11].

Two manuscripts are respectively related to factors associated with early detection of HIV among children of HIV-infected FSW in Cameroon and partner notification in Botswana. In Cameroon, out of the 481 FSW interviewed in the study of Rao et al., 70% reported that none of their children under the age of 5 years had been tested for HIV. The factors influencing HIV testing of the children of HIV-positive FSW were antenatal services attendance (OR adjusted 2.12, 95% CI: [1.02, 4.55]), knowing HIV status (OR 3.70 [2.30, 5.93]), the character desired of the pregnancy (OR 1.89 [1.16, 3.08]) and higher education level (OR 2.17 [1.01, 4.71]). Partner notification and proper treatment of partners are essential elements for breaking the chain of transmission of sexual transmissible infections (STI) including HIV infection. The supplement includes one qualitative study done by A. Wynn et al. (2019), in Botswana. The study revealed that treatment of partners was late and most participants expressed a preference for notifying their STI to their partners at a health facility with support from the health workers. The authors concluded that important progress remain to be made along the four pillars of PMTCT especially among key populations such as FSW [12, 13].

Cervical cancer is one of the leading causes of cancer deaths in women especially in middle and low income countries. It is associated with persistent or high-risk (or oncogenic) types of human papillomavirus (HPV). Its prevalence is higher among certain vulnerable groups like people living with HIV. This supplement includes an original study on the feasibility in rural areas of Zimbabwe to integrate HPV screening within existing community-based HIV programming and immunization outreach services. Samples were collected at community level by trained community health workers. The collection was performed during scheduled outreach visits for antiretroviral medications and childhood vaccines. Community health workers explained how to perform the self-collection sample. The samples were then transferred to a health facility for analysis. This integrated community outreach model was accepted by the beneficiaries with an 82% participation rate [14].

## Conclusion

Ending AIDS as a public health threat and ensuring universal access to sexual and reproductive health are two key targets for Sustainable Development Goal (SDG) 3. The articles reinforce that as we journey towards 2030, integration of SRHR and HIV can play an important role in improving the health and well-being for all. As Narasimhan et al. remind us global progress and success for reaching the SRHR targets depend on progress being made in sub-Saharan Africa that has the highest burden of unintended pregnancies, STIs including HIV.

A key message emerging from the collection of articles is the need for the principle of bi-directionality to be applied in all SRHR and HIV interventions. These articles reinforce one of the key action points in the *Call to action to attain universal health coverage through linked SRHR and HIV interventions* issued at the International AIDS Conference in 2018. This call emphasizes that for interventions to have the desired impact they need to meaningfully engage communities in the design, implementation and monitoring in order to meet the community’s needs.

A common thread is that integration can only be effective if investments are made in building the capacity of health care workers, and ensuring that health facilities have the necessary infrastructure, are well equipped and have adequate commodities supply. It is also evident that integration efforts should include not only health facilities but also community outreach interventions.

While the SDGs call on the global community to “leave no one behind”, efforts need to be redoubled to address the needs of key and vulnerable populations within the four prongs of PMTCT and adolescent girls and young women within treatment, and care services in emergency settings. This collection of articles demonstrates that bringing together SRHR and HIV will require deliberate interventions and political commitment that places the individual at the center of service delivery.

A French translation of this article has been included as Additional file 1.

A Portuguese translation of this article has been included as Additional file 2.

## Additional files

Additional file 1: Translation of this article into French. (PDF 173 kb)

Additional file 2: Translation of the abstract of this article into Portuguese. (PDF 451 kb)

## Author’s information

Dr. Didier Mbayi Kangudie, MD, MPH (mkangudie@usaid.gov) is Senior Health Advisor, USAID/West Africa, Regional Health Office. Dr. Hugues Guidigbi, MD, MPH (hguidigbi@usaid.gov) is Senior HIV/AIDS Advisor, USAID/West Africa, Regional Health Office. Sheila Mensah (smensah@usaid.gov) is Senior Communications, Monitoring & Evaluation Advisor, USAID/West Africa, Regional Health Office. Abdul A. Bala (abala@usaid.gov) is Program and Research Analyst, USAID/West Africa, Regional Health Office. Richard Delate (delate@unfpa.org) is Program Specialist SRH/HIV, UNFPA, East and Southern Africa Regional Office.
