# Supplementary material for: Effective integration of sexual reproductive health and HIV prevention, treatment, and care services across sub-Saharan Africa: where is the evidence for program implementation?
Source: Reprod Health. 2019 May 29;16(Suppl 1):56. doi: 10.1186/s12978-019-0709-6 (PMC6538537; doi:10.1186/s12978-019-0709-6)
Supplement: Supplementary file 1 — Translation of this article into French. (PDF 173 kb) [file 12978_2019_709_MOESM1_ESM.pdf]

Reproductive Health 2019, 16 (Suppl 1) [translated from the original publication]

Intégration efficace des Services de Santé sexuelle et reproductive et des Services de Prévention, de Traitement et de Soins liés au VIH en Afrique subsaharienne : Où est la preuve de la mise en œuvre du programme ?

Didier Mbayi Kangudie[1], Hugues Guidigbi [1], Sheila Mensah[1], Abdul A. Bala[1], Richard Delate[2]*

Affiliations des auteurs
[1] United States Agency for International Development, West Africa Mission, Regional Health Office
[2] United Nations Population Fund East and Southern Africa Regional Office

*Auteur correspondant : Didier Mbayi Kangudie mkangudie@usaid.gov

Introduction

Mettre fin au sida en tant que menace pour la santé publique tout en visant un accès universel aux services de Santé sexuelle et reproductive (SSR) reste un objectif stratégique double des programmes VIH/SIDA et SSR. La réalisation de cet objectif dans les deux programmes nécessitera des interventions mûrement étudiées pour harmoniser la mise en œuvre aux niveaux politique, des systèmes, du financement, de la coordination, de la gestion, de la prestation des services et de la surveillance.

En 2017, 25,6 millions de personnes vivaient avec le VIH en Afrique subsaharienne [4]. Cela représente 69,6 % des personnes vivant avec le VIH dans le monde. Le nombre de nouvelles infections chez les enfants reste élevé avec 159 000 nouvelles infections en 2017, malgré les progrès réalisés au cours des deux dernières décennies pour éliminer la transmission du VIH de la mère à l'enfant [4 ; 7 ; 8]. L'enquête communautaire mondiale menée en 2014 sur les priorités de la SSR visant à informer les femmes vivant avec le VIH des directives de l'OMS a révélé un taux de grossesse non planifiée de 56,7 %, dont seulement 55,3 % des femmes vivant avec le VIH ayant bénéficié d'un soutien pratique pour utiliser des méthodes de conception sûres. Dans trois grandes maternités de l'est du Cap, en Afrique du Sud, la prévalence des grossesses non planifiées chez les femmes vivant avec le VIH était de 71 % (Oladele Adeniyi *et al.*) [11].

En outre, le taux de prévalence des méthodes de contraception modernes est faible en Afrique subsaharienne, avec une estimation de 28 % [2], et les besoins non satisfaits en matière de services de planning familial (PF) restent élevés, à 22 %, soit près du double de la moyenne mondiale [3]. Pour mettre fin au sida et assurer l'accès universel aux services de santé sexuelle et reproductive, y compris au PF, d'ici 2030, il faudra faire des choix d'investissement stratégiques et adopter des modèles d'intégration intelligents.

Depuis l'appel à l'action 2004 de Glion sur le lien entre le PF et la prévention de la transmission du VIH de la mère à l'enfant (PTME), plusieurs initiatives et politiques ont été mises en place pour faciliter l'intégration des services liés à la SSR et au VIH [5]. L'intégration des services fait depuis longtemps l'objet de débats. Johnson, Varallyay et Ametepi (2018) ont abordé cette question en proposant une revue de la littérature qui montrait que le lexique des termes faisant référence à des concepts liés à « l'intégration » variait et que sa définition n'était pas comprise de manière uniforme. Le terme apparenté le plus couramment utilisé, parfois de manière interchangeable, est le concept de « liens ». L'Organisation mondiale de la santé a tenté de faire la distinction entre ces deux termes, en définissant les « liens » comme un concept englobant plus largement les synergies existant

entre les politiques, les programmes, les services et les efforts de plaidoyer relatifs à la santé sexuelle et reproductive et au VIH, tout en définissant « l'intégration » comme un niveau plus spécifiquement axé sur des services ciblés et/ou des programmes pouvant être combinés pour assurer et maximiser les résultats collectifs en offrant des services plus complets. Cette définition nécessite des structures organisationnelles et des procédures de gestion spécifiques pour favoriser cette amélioration de la prestation de services [1].

Malgré l'insuffisance de données permettant d'évaluer pleinement les effets de l'intégration des services de SSR et droits/VIH sur les grossesses non planifiées en comparant les résultats des sites intégrés aux sites non intégrés, plusieurs études ont montré une réduction des coûts de prestation des services, une meilleure connaissance de la patientèle et une utilisation supérieure des méthodes contraceptives modernes (Haberlen, Narasimhan, Beres & Kennedy, 2017) [30]. Pour atteindre des niveaux d'efficacité opérationnelle supérieurs, les responsables de projet peuvent tirer des enseignements des modèles d'intégration de services documentés et réussis. Comme l'ont reconnu Johnson, Varallyay et Ametep (2018), les recommandations des gouvernements, des donateurs, des organisations internationales et des organismes de normalisation soutiennent explicitement l'intégration de la prestation de services liés au VIH et à la santé reproductive, en particulier le planning familial. Il est reconnu que l'intégration est nécessaire pour répondre aux besoins des femmes et des hommes en matière de santé reproductive, pour faire reculer l'épidémie de VIH dans le but de mettre fin au sida en tant que menace pour la santé publique et pour assurer un accès universel aux services de santé sexuelle et reproductive d'ici à 2030, conformément aux Objectifs de développement durable (ODD) [1].

La compilation d'articles dans ce supplément aborde l'intégration des services de SSR et VIH/SIDA sous différents angles. De l'intégration du planning familial en tant que deuxième volet de la prévention de la transmission verticale du VIH, y compris chez les travailleuses du sexe, au concept de grossesse à moindre risque chez les personnes touchées par le VIH, en passant par l'intégration de la SSR dans l'ensemble de services minimum de prévention, de soins et de traitement du VIH. Les articles examinent également la conception d'approches novatrices d'intégration dans les modèles de prestation de services communautaires. Il est difficile de définir le succès de l'intégration des services et les expériences partagées dans ces articles couvrent plusieurs pays et zones géographiques, notamment au Botswana, au Cameroun, au Kenya, au Malawi, en Afrique du Sud, en Tanzanie, en Ouganda et aux États-Unis. En outre, une analyse systématique et une analyse de la littérature existante explorent respectivement la question spécifique de l'intégration du dépistage du VIH dans le planning familial et la question de l'intégration de la santé sexuelle et reproductive des adolescentes et des jeunes femmes dans les situations d'urgence.

Discussion

Une intégration efficace des services liés au VIH et à la santé sexuelle et reproductive exige non seulement des interventions visant à modifier le comportement des prestataires de soins de santé (Changement de comportement des prestataires), mais également une meilleure compréhension des bénéficiaires pour répondre de manière appropriée à leurs besoins en fonction de leurs connaissances, de leur attitude et de leur perception du risque. Comme démontré par González, Kadengye & Mayega (2019) dans leur enquête représentative auprès des foyers, à l'échelle nationale et axée sur les jeunes Ougandais, des

niveaux élevés de connaissance de la SSR/du VIH et de la perception du risque n'ont pas permis de freiner les comportements sexuels à risque, malgré le contexte d'épidémie généralisée de VIH (prévalence de 2,1 % parmi les 15-24 ans). Les auteurs ont affirmé que les lacunes en matière d'efficacité dans la réponse intégrée SSR/VIH devraient être traitées de manière globale, aux niveaux individuel et structurel [14].

Dans ce supplément, l'attention a notamment été portée sur un groupe marginalisé parmi les populations clés d'Afrique subsaharienne, les femmes qui s'injectent des drogues. Sylvia Ayon *et al.* (2019) ont utilisé la recherche-action pour identifier le processus, les impacts et les défis liés à l'intégration de la SSR dans les programmes de prévention du VIH et de réduction des risques pour la communauté dans les villes côtières du Kenya. Leurs conclusions mettent en évidence la faible utilisation des services de planning familial et d'autres services de santé sexuelle et reproductive, et fournissent des informations essentielles en matière d'acceptabilité et de possibilités d'intégration réussie au niveau communautaire [25].

Une étude transversale réalisée au Kenya par Raymond Mutisya *et al.* a évalué le niveau d'intégration du planning familial dans six autres domaines de prestation de services (CPN, maternités, centre de soins postnataux, centre de protection de l'enfance, conseil et dépistage du VIH, services de lutte contre le VIH/SIDA dans des centres de soins complets). Leurs résultats confirment ce qui a déjà été documenté sur la corrélation positive entre connaissances, compétences et attitudes du prestataire et qualité des services [21].

L'analyse « A systematic review of the integration of HIV testing services into family planning services » par Narasimhan *et al.* (2019) a révélé que les services de conseil et de dépistage du VIH étaient généralement plus élevés dans les sites intégrés que dans les sites non intégrés, y compris dans les analyses ajustées en fonction des résultats, légèrement différents d'une étude à l'autre. Comme dans les conclusions de Kiersten Johnson *et al.*, cette analyse conclut que les progrès et les succès globaux dans la réalisation des objectifs de SSR et de VIH dépendent des progrès accomplis en Afrique subsaharienne, où les femmes supportent un lourd fardeau tant de grossesses non désirées que d'infections sexuellement transmissibles, y compris le VIH [22].

Dans un rapport sur l'état du monde de 2015, le FNUAP a déclaré que les nombreuses crises, guerres et catastrophes naturelles dans le monde, et en particulier en Afrique, exposent les femmes et les adolescentes à un risque considérablement accru de grossesse non désirée, de décès maternel, de violence sexuelle et de contraction du VIH. Ce supplément comprend seulement une analyse documentaire de Roxo, Walker, Mobula, Ficht & Yeiser (2019) sur la priorité accordée à la SSR et aux droits des adolescentes et des jeunes femmes dans les services de traitement et de soins en situation d'urgence. Leur analyse a révélé que la pluralité des besoins en concurrence dans les situations d'urgence libère du temps et de l'espace consacrés à l'intégration efficace des interventions relatives au VIH et à la SSR et qu'une plus grande volonté politique est nécessaire pour faire avancer le programme d'intégration [26].

Deux des articles du supplément traitent des principaux résultats concernant la disponibilité de services intégrés de planning familial et liés au VIH en Afrique subsaharienne, l'un ayant

trait à la composante qualité. Des études menées par Kanyangarara, Sakyi & Laar (2019) et Barden-O'Fallon, Mejia & Close (2019) ont limité les analyses aux établissements de santé proposant des soins et un soutien en matière de VIH/SIDA, et pas seulement des services de conseil et de dépistage du VIH et de PF. À l'aide d'une analyse secondaire des Évaluations de prestations de services (SPA) et des Évaluations de la disponibilité et de la capacité opérationnelle des services (SARA) (2012 - 2015) pour 10 pays, dont six en Afrique de l'Ouest et du Centre), Kanyangara *et al.* ont constaté que 93 % des établissements offrant des services de prise en charge et de soins du VIH indiquaient également offrir des services intégrés, mais que 29 % seulement étaient classés comme ayant des services de PF intégrés sur place, en fonction de la disponibilité d'intrants structurels et de traitement (p. ex. équipement, directives, prestataires formés et produits de PF). En outre, 94 % des établissements déclaraient offrir systématiquement des services de conseil en PF aux clientes atteintes du VIH/SIDA et 80 % avaient trois méthodes de contraception ou plus en stock au moment des enquêtes [23, 29].

En comparant les mesures de SPA et les indicateurs QIQ (Enquête rapide sur la qualité) des services de PF dans des structures de soins et de soutien de niveau inférieur intégrées aux services liés au VIH/SIDA au Malawi (2013 - 2014) et en Tanzanie (2014 - 2015), Close *et al.* ont constaté que 79 % des établissements au Malawi et 38 % des établissements en Tanzanie offrent des services de PF. Conformément au cadre de qualité des soins de Bruce/Jain, 22 indicateurs de qualité ont été analysés et ont montré que le statut d'intégration était fortement lié à trois indicateurs pour le Malawi : « le centre dispose de toutes les méthodes (approuvées) disponibles : pas de rupture de stock », « le centre a reçu une visite de contrôle au cours des 6 derniers mois », « consultation et annotation du dossier du patient ». Pour la Tanzanie, les indicateurs associés au statut d'intégration étaient les suivants : « le centre dispose d'un stock adéquat de contraceptifs et de médicaments », « le centre dispose de toutes les méthodes (approuvées) disponibles : pas de rupture de stock », « le temps d'attente est acceptable (inférieur) », et « la structure dispose des mécanismes nécessaires pour apporter des modifications de programme en fonction des commentaires des patients » [23, 29].

Deux des articles ont analysé les connaissances et l'utilisation de méthodes de conception plus sûres chez les personnes infectées par le VIH pour répondre à leurs besoins en la matière. Les deux études de Gwokyalya *et al*. (2019) et Schwartz *et al*. (2019) ont souligné la nécessité d'une éducation accrue et de la mise à disposition de différentes méthodes de gestion de la chaîne d'approvisionnement pour les personnes vivant avec le VIH. Sur les 5198 femmes interrogées dans les centres de santé en Ouganda, 74,1 % avaient une connaissance des MSC, mais ce nombre diminue à 42 % parmi celles connaissant plusieurs méthodes. L'étude a également souligné le manque d'implication des partenaires ayant des relations sérodiscordantes dans le choix et l'intention d'utiliser les MSC. Ils ont insisté sur le manque de personnel qualifié pour entreprendre certaines MSC, telles que le lavage du sperme. L'étude a mis en évidence les défis croissants posés par les personnes vivant avec le VIH pour réaliser leur désir de fécondité et a conclu que la connaissance et l'utilisation des MCS chez les femmes séropositives prises en charge sont faibles. Les efforts visant à améliorer la divulgation du VIH, l'intégration d'une conception plus sûre dans les services de PF et de VIH et les efforts régionaux visant à promouvoir la sensibilisation et l'accès à une

conception plus sûre peuvent contribuer à accroître l'utilisation de méthodes de conception plus sûres [24, 28].

Grâce à son approche en quatre volets, la PTME prévient 90 % des nouvelles infections de VIH chez les enfants et contribue ainsi aux efforts visant à créer une génération sans sida. La PTME est également à la croisée des chemins des services de soins prénataux et postnataux, du PF et de la prévention du VIH. Trois manuscrits de ce supplément traitent de la question de la PTME du point de vue de l'intégration des services prénataux, liés au VIH et de santé sexuelle et reproductive. Les auteurs, Rwema *et al*. (2019), et Parmley *et al*. (2019) ont évalué l'approche en cascade de la PTME et étudié les facteurs influençant les services de soins prénataux à la recherche d'un comportement dans un contexte de prévalence élevée du VIH chez les travailleuses du sexe à Port Elizabeth, en Afrique du Sud [15, 16].

Rwema *et al.* (2019) ont constaté que 61 % des travailleuses du sexe (FSW) étaient infectées par le VIH et que 52 % d'entre elles savaient qu'elles étaient atteintes du VIH avant l'étude. Un écart de 40 % a été constaté concernant l'utilisation systématique de préservatifs par les FSW non-infectées par le VIH avec leurs clients et un écart de 43 % dans l'utilisation de méthodes contraceptives modernes à long terme chez les FSW vivant avec le VIH. Parmley *et al.* (2019) ont constaté, dans un contexte similaire à Port Elizabeth, en Afrique du Sud, une découverte tardive de grossesse (entre 4 et 7 mois) parmi les FSW vivant avec le VIH et une couverture de traitement antirétroviral de 40 % d'entre elles. Les facteurs d'influence identifiés étaient la consommation d'alcool et de substances psychoactives, ainsi que l'insatisfaction par rapport aux expériences antérieures en matière de soins de santé [15, 16].

Deux manuscrits sont respectivement liés à des facteurs associés à la détection précoce du VIH chez les enfants de FSW vivant avec le VIH au Cameroun et à la notification de partenaires au Botswana. Au Cameroun, sur les 481 FSW interrogées dans l'étude de Rao *et al.*, 70 % ont déclaré qu'aucun de leurs enfants de moins de 5 ans n'avait subi de test de dépistage du VIH. Les facteurs influençant le test de dépistage du VIH chez les enfants de FSW séropositives étaient la fréquentation des services prénataux (OR ajusté 2,12, IC 95 % : [1,02, 4,55]), la connaissance de la séropositivité (OR 3,70 [2,30, 5,93]), le caractère souhaité de la grossesse (OR 1,89 [1,16, 3,08]) et le niveau de scolarisation supérieur (OR 2,17 [1,01, 4,71]). La notification des partenaires et le traitement approprié des partenaires sont des éléments essentiels pour rompre la chaîne de transmission des infections sexuellement transmissibles (IST), notamment l'infection du VIH. Le supplément comprend une étude qualitative réalisée par A. Wynn *et al.* (2019), au Botswana. L'étude a révélé que le traitement des partenaires était tardif et la plupart des participants ont indiqué préférer informer leurs partenaires de leur IST dans un établissement de santé avec le soutien des agents de santé. Les auteurs ont conclu que des progrès importants restaient à accomplir selon les quatre piliers de la PTME, en particulier parmi les populations clés telles que les FSW [17, 18].

Le cancer du col utérin est l'une des principales causes de décès par cancer chez les femmes, en particulier dans les pays en voie de développement à revenu faible et intermédiaire. Il est associé aux types de papillomavirus humain (PVH) persistants ou à haut risque (ou oncogènes). Sa prévalence est plus élevée chez certains groupes vulnérables comme les personnes vivant avec le VIH. Ce supplément comprend une étude originale sur la faisabilité, dans les zones rurales du Zimbabwe, d'intégrer le dépistage du PVH dans les programmes communautaires existants de lutte contre le VIH et les services de proximité en

matière de vaccination. Les échantillons ont été prélevés au niveau communautaire par des agents de santé communautaires formés. Le prélèvement a été effectué lors de visites de sensibilisation prévues pour les médicaments antirétroviraux et les vaccins pour enfants. Les agents de santé communautaires ont expliqué comment réaliser l'auto-prélèvement d'échantillon. Les échantillons ont ensuite été transférés dans un établissement de santé pour analyse. Ce modèle de proximité intégré a été accepté par les bénéficiaires avec un taux de participation de 82 % [20].

Conclusion

Mettre fin au sida en tant que menace pour la santé publique et garantir l'accès universel aux services de santé sexuelle et reproductive sont deux objectifs clés de l'Objectif de développement durable (ODD) 3. Les articles insistent sur le fait que, à l’horizon 2030, l’intégration des services de SDSR et de VIH peut jouer un rôle important dans l’amélioration de la santé et du bien-être de tous. Comme Narasimhan *et al.* nous le rappelle, les progrès et les succès mondiaux dans la réalisation des objectifs de SDSR dépendent des progrès réalisés en Afrique subsaharienne, qui est le pays le plus touché par les grossesses non désirées, les IST, notamment le VIH.

L'un des messages clés ressortant de l'ensemble des articles, c'est la nécessité d'appliquer le principe de bidirectionnalité dans toutes les interventions de SDSR et de lutte contre le VIH. Ces articles renforcent l’un des points essentiels de l’*Appel à l’action pour atteindre la couverture sanitaire universelle par le biais d’interventions liées à la santé sexuelle et reproductive et au VIH* (*Call to action to attain universal health coverage through linked SRHR and HIV interventions*) lancé à la Conférence internationale sur le sida en 2018. Cet appel met l'accent sur un point essentiel : pour que les interventions produisent l’impact souhaité, elles doivent faire participer de manière significative les communautés à la conception, à la mise en œuvre et au suivi des programmes afin de répondre à leurs besoins.

En tant que fil conducteur, l'intégration ne peut être efficace que si des investissements sont consentis pour renforcer les capacités des personnels de santé et veiller à ce que les établissements de santé disposent des infrastructures nécessaires, soient bien équipés et disposent de suffisamment de produits de base. Il est également évident que les efforts d'intégration devraient inclure non seulement les établissements de santé, mais également les interventions de proximité.

Alors que les ODD appellent la communauté mondiale à « ne laisser personne derrière », il faut redoubler d'efforts pour répondre aux besoins des populations clés et vulnérables dans les quatre domaines de la PTME et des adolescentes et jeunes femmes sous traitement, ainsi que des services de soins dans les contextes d'urgence. Cet ensemble d'articles démontre que la réunion des services de SDSR et de VIH nécessitera des interventions mûrement étudiées et un engagement politique qui place l'individu au centre de la prestation de services.

Références

1. Kiersten Johnson, Ilona Varallyay, Paul Ametepi. Integration of HIV and Family Planning Health Services in Sub-Saharan Africa: A Review of the Literature, Current Recommendations, and Evidence from the Service Provision Assessment Health Facility Surveys, ICF International, USA, 2018.
2. PRB, World Population Data Sheet, 2018.
3. United Nations, World Family Planning, 2017.
4. UNAIDS. Data 2018. Geneva: UNAIDS; 2018. http://www.unaids.org/sites/default/files/media_asset/unaids-data-2018_en.pdf.
5. The Glion Call to Action on Family Planning and HIV/AIDS in Women and Children 3-5 May 2004.
6. UNFPA 2016. Universal access to reproductive health. Progress and challenges. Geneva: UNFPA January 2016, p 16.
7. Hladik W, Stover J, Esiru G, Harper M, Tappero J (2009). The Contribution of Family Planning towards the Prevention of Vertical HIV Transmission in Uganda. PLoS ONE 4(11): e7691. doi:10.1371/journal.pone.0007691.
8. Vrazo AC, Sullivan D, Ryan Phelps B. Eliminating mother-to-child transmission of HIV by 2030: 5 strategies to ensure continued progress. Glob Health Sci Pract. 2018; 6 (2) : 249-256. https://doi.org/10.9745/GHSP-D-17-00097.
9. WHO. Preventing HIV and Unintended Pregnancies: Strategic Framework 2011–2015. Geneva: World Health Organization 2003, p85.
10. WHO. Strategic approaches to the prevention of HIV infection in infants: report of a WHO meeting, Morges, Switzerland, 20-22 March 2002. Geneva: World Health Organization 2003, p10.
11. Oladele Vincent Adeniyi, Anthony Idowu Ajayi, Mayowa Gabriel Moyaki, Daniel Ter Goon, Gordana Avramovic and John Lambert. High rate of unplanned pregnancy in the context of integrated family planning and HIV care services in South Africa. BMC Health Services Research (2018) 18:140. https://doi.org/10.1186/s12913-018-2942-z.
12. WHO. Preventing HIV and Unintended Pregnancies: Strategic Framework 2011–2015. Geneva: World Health Organization 2003.
13. Vrazo AC, Sullivan D, Ryan Phelps B. Eliminating mother-to-child transmission of HIV by 2030: 5 strategies to ensure continued progress. Glob Health Sci Pract. 2018; 6 (2) : 249-256. https://doi.org/10.9745/GHSP-D-17-00097.
14. P.R. González, D.T. Kadengye, R.W Mayega. The Knowledge-Risk-Behaviour Continuum among Young Ugandans: What it tells us about SRH/HIV Integration. BMC Public Health. 2019; 19 Suppl 1:S2
15. J. O. T. Rwema, S. Baral, S. Ketende, N. Phaswana-Mafuya, A. Lambert, Z. Khose, M. Mcingana, A. Rao, H. Hausler, S. Schwartz. Evaluating the Vertical HIV Transmission Risks and PMTCT cascades among South African Female Sex Workers. Have we forgotten PMTCT in their HIV programming? BMC Public Health. 2019; 19 Suppl 1:S3

16. L. Parmley, A. Rao, Z. Kose, A. Lambert, R. Max, N. Phaswanamafuya, M. Mcingana, H. Hausler, S. Baral, S. Schwartz. Antenatal care presentation and engagement in the context of sex work: Exploring barriers to care for sex worker mothers in South Africa. Reproductive Health. 2019; 16 Suppl 1:S4
17. A. Rao, S. Schwartz, S. C. Billong, A. Bowring, G. Fouda, F. Ndonko, I. Njindam, D. Levitt, A-C. Bissek, O. Njoya, S. Baral. Predictors of Early Childhood HIV Testing among Children of Sex Workers Living with HIV in Cameroon. BMC Public Health. 2019; 19 Suppl 1:S4
18. A. Wynn, C. Moucheraud, N. Moshashane, O. A. Offorjebe, D. Ramogola-Masire, J. D Klausner, C. Morroni. Using partner notification to address curable sexually transmitted infections in a high HIV prevalence context: A qualitative study about partner notification in Botswana. BMC Public Health. 2019; 19 Suppl 1:S5
19. N. Broutet, L. O'Neal Eckert, A. Ullrich, P. Bloem. Comprehensive cervical cancer control. A guide to essential practice. Geneva: World Health Organization 2014.
20. M. B. Fitzpatrick, Z. El-Khatib, D, Katzenstein, B. A. Pinsky, Z. M. Chirenje, K. McCarty. Community-Based Self-Collected Human Papillomavirus Screening in Rural Zimbabwe. BMC Public Health. 2019; 19 Suppl 1:S2
21. R. Mutisya, S. Karnad, J. Wambua, M. Kabue, F. Waweru, E. Omanga. Strengthening integration of family planning with HIV services: The Tupange project experience in three Kenyan cities. Reproductive Health. 2019; 16 Suppl 1:S6
22. M. Narasimhan, P. Teresa Yeh, S. Haberlen, C. E. Warren, C. E. Kennedy. Integration of HIV testing services (HTS) into family planning (FP) services: a systematic review. Reproductive Health. 2019; 16 Suppl 1:S5
23. J. Barden-O'Fallon, C. Mejia, M. A. Close. Quality of family planning services of HIV integrated and non-integrated facilities in Malawi and Tanzania. Reproductive Health. 2019; 16 Suppl 1:S3
24. S. Schwartz, N. Davies, N. Naidoo, D. Pillay, N. Sikhosana, S. Mullick. Clients' experiences utilizing a safer conception service for HIV affected individuals: Implications for differentiated care service delivery models. Reproductive Health. 2019; 16 Suppl 1:S9
25. S. Ayon, F. Jeneby, F. Hamid, A. Badhrus, T. Abdulrahman, G. Mburu. Developing integrated community-based HIV prevention, harm reduction, and sexual and reproductive health services for women who inject drugs. Reproductive Health. 2019; 16 Suppl 1:S2
26. U. Roxo, D. Walker, Linda Mobula, Allison Ficht, Sarah Yeiser. Prioritizing the Sexual Reproductive Health and Rights of Adolescent Girls and Young Women within HIV Treatment and Care Services in Emergency Settings: A Girl-Centered Agenda. Reproductive Health. 2019; 16 Suppl 1:S1
27. V. Gwokyalya, J. Beyeza-Kashesya, J. B. Bwanika, J. KB Matovu, S. Mugerwa, J. Arinaitwe, D. Kasozi, J. Bukenya, R. Kindyomunda, G J. Wagner, F E. Makumbi and R K. Wanyenze. Knowledge and correlates of use of safer conception methods among HIV-infected women attending HIV care in Uganda. Reproductive Health. 2019; 16 Suppl 1:S8

28. M. Kanyangarara, K. Sakyi, A. Laar. Availability of integrated family planning services in HIV care and support sites in sub-Saharan Africa: a secondary analysis of national health facility surveys. Reproductive Health. 2019; 16 Suppl 1:S4
29. Sabina A. Haberlen, Manjulaa Narasimhan, Laura K. Beres, and Caitlin E. Kennedy. Integration of Family Planning Services into HIV Care and Treatment Services: A Systematic Review. Studies in Family Planning, 2017.

Concernant ce supplément

Cet article a été publié comme partie de l'ouvrage de *BMC Public Health*, Volume 19 Supplement 1, 2019: Effective Integration of Sexual Reproductive Health and HIV Prevention, Treatment, and Care Services across sub-Saharan Africa: Where is the evidence for program implementation?
Le supplément a été publié dans le cadre d'une collaboration entre *Reproductive Health* et *BMC Public Health.* L'intégralité du contenu, avec les versions en français, en portugais et en anglais, est disponible en ligne :
https://bmcpublichealth.biomedcentral.com/articles/supplements/volume-19-supplement-1
et
https://reproductive-health-journal.biomedcentral.com/articles/supplements/volume-16-supplement-1

Déclarations

Approbation éthique et accord de participation
Non applicable

Accord de publication
Non applicable

Disponibilité des données et matériels
Non applicable

Conflits d'intérêts
Les auteurs déclarent ne pas avoir de conflits d'intérêts.

Financement
Le supplément de la revue est rendu possible grâce au soutien généreux du peuple américain via la United States Agency for International Development (USAID) en partenariat avec le Fonds des Nations unies pour la population (FNUAP) et le Programme commun des Nations Unies sur le VIH/SIDA (ONUSIDA).
Les opinions exprimées dans la présente publication sont celles des auteurs et ne reflètent pas nécessairement les politiques officielles de l'USAID, du FNUAP ou de l'ONUSIDA, la mention des dénominations de ministères ou d'organismes n'implique pas non plus l'aval du gouvernement américain, du FNUAP ou de l'ONUSIDA.

Contributions des auteurs
DMK a contribué à l'introduction de l'article et à la discussion sur les manuscrits liés à l'intégration des services de SSR et de VIH de manière générale. HG a contribué à l'introduction et à la discussion sur des manuscrits liés aux travailleuses du sexe et à l'intégration des soins prénataux/services liés au VIH. SM a contribué à la discussion sur les manuscrits liés à la disponibilité et à la qualité de l'intégration des services de SSR et de VIH. AB a contribué à la discussion sur les manuscrits liés à la sécurité de la conception et aux PVVIH. RD a contribué à la conclusion. Tous les auteurs ont lu et approuvé l'article final.

Remerciements
Le comité de rédaction du Supplément tient à exprimer sa profonde gratitude envers tous les pairs examinateurs pour leur temps et leur expertise. Le comité souhaite également mettre en avant les contributions importantes de Susan Mathew, Jennifer Mason, Nithya Mani et Sheryl Martin au cours des différentes étapes du processus d'évaluation par les pairs et de la révision finale de cet article.

Les opinions exprimées dans la présente publication sont celles des auteurs et ne reflètent pas nécessairement les politiques officielles de l'USAID, du FNUAP ou de l'ONUSIDA, la mention des dénominations de ministères ou d'organismes n'implique pas non plus l'aval du gouvernement américain, du FNUAP ou de l'ONUSIDA.

Informations sur les auteurs

Le Dr Didier Mbayi Kangudie, MD, MPH (mkangudie@usaid.gov) est Conseiller principal pour la santé, USAID/Afrique de l'Ouest, Bureau régional de la santé. Le Dr Hugues Guidigbi, MD, MPH (hguidigbi@usaid.gov) est Conseiller principal pour le VIH/SIDA, USAID/Afrique de l'Ouest, Bureau régional de la santé. Sheila Mensah (smensah@usaid.gov) est Conseillère principale en Communications, Contrôles et Évaluations, USAID/Afrique de l'Ouest, Bureau régional de la santé. Abdul A. Bala (abala@usaid.gov) est Analyste de Programme et Recherche, USAID/Afrique de l'Ouest, Bureau régional de la santé. Richard Delate (delate@unfpa.org) est Spécialiste du Programme SSR/VIH, FNUAP, Afrique de l'Est et du Sud, Bureau régional
