# Supplementary material for: Effective integration of sexual reproductive health and HIV prevention, treatment, and care services across sub-Saharan Africa: where is the evidence for program implementation?
Source: Reprod Health. 2019 May 29;16(Suppl 1):56. doi: 10.1186/s12978-019-0709-6 (PMC6538537; doi:10.1186/s12978-019-0709-6)
Supplement: Supplementary file 2 — Translation of the abstract of this article into Portuguese. (PDF 451 kb) [file 12978_2019_709_MOESM2_ESM.pdf]

Integração Eficaz dos Serviços de Saúde Sexual e Reprodutiva e de Prevenção, Cuidados e Tratamento do VIH na África Subsariana: onde estão as provas da implementação do programa?

Didier Mbayi Kangudie[1], Hugues Guidigbi [1], Sheila Mensah[1], Abdul A. Bala[1], Richard Delate[2]*

Afiliações dos autores
[1] United States Agency for International Development, West Africa Mission, Regional Health Office
[2] United Nations Population Fund East and Southern Africa Regional Office

*Autor correspondente: Didier Mbayi Kangudie mkangudie@usaid.gov

Introdução

Pôr fim à SIDA como uma ameaça para a saúde pública, visando em simultâneo o acesso universal à Saúde Sexual e Reprodutiva (SSR), continua a ser um objetivo estratégico duplo da programação da luta contra o VIH/SIDA e da SSR. A promoção deste objetivo nas duas áreas de programação exigirá intervenções deliberadas para interligar a implementação aos níveis político, de sistemas, financiamento, coordenação, gestão, prestação de serviços e monitorização.

Em 2017, 25,6 milhões de pessoas viviam com VIH na África Subsariana [4]. Este número representa 69,6% das pessoas que vivem com VIH no mundo. O nível de novas infeções entre as crianças continua elevado, tendo sido registadas 159.000 novas infeções em 2017, apesar dos progressos realizados durante as últimas duas décadas para eliminar a transmissão mãe-filho do VIH [4; 7; 8]. O estudo da comunidade global realizado em 2014 sobre as prioridades da SSR entre as mulheres que vivem com VIH, para elaboração das diretrizes da OMS, revelou uma taxa de gravidez não planeada de 56,7%, tendo apenas 55,3% das mulheres que vivem com VIH beneficiado de apoio prático relativamente a métodos de conceção seguros. Em três maternidades de grande dimensão no Cabo Oriental, na África do Sul, a prevalência da gravidez não planeada entre as mulheres que vivem com VIH era de 71% (Oladele Adeniyi *et al.*) [11].

Além disso, a África Subsariana tem uma taxa de prevalência de contracetivos modernos reduzida, estimada em 28% [2], e uma elevada necessidade continuada, por satisfazer, de planeamento familiar (PF) de 22%, quase o dobro da média global [3]. O caminho para pôr fim à SIDA e garantir o acesso universal à SSR, incluindo ao PF, até 2030, irá exigir escolhas estratégicas de investimento e modelos de integração inteligentes.

Desde o Apelo à Ação em Glion, em 2004, sobre a ligação entre o PF e a Prevenção da Transmissão mãe-filho do VIH (PTV), foram implementadas várias iniciativas e políticas para facilitar a integração da SSR e do VIH [5]. O que constitui a integração dos serviços é há muito objeto de debate. Johnson, Varallyay e Ametepi (2018) abordaram o tema com uma revisão bibliográfica que revelou que o léxico de termos que se referem a conceitos relacionados com "integração" é variado e a sua definição não é compreendida de forma uniforme. O termo relacionado mais utilizado, por vezes de forma indistinta, é o conceito de "articulação". A Organização Mundial da Saúde tentou distinguir os dois termos, definindo

"articulação" como um conceito que engloba de forma mais ampla as sinergias existentes entre as políticas, programas, serviços e esforços de sensibilização para a saúde sexual e reprodutiva e o VIH, e definindo "integração" como um nível focado mais especificamente nos serviços e/ou programas adequados que podem ser combinados para garantir e maximizar os resultados coletivos através da oferta de serviços mais abrangentes. Tal definição exige procedimentos de gestão e estruturas organizacionais específicas para suportar esta prestação melhorada de serviços [1].

Apesar de não existirem dados suficientes para avaliar cabalmente o efeito da integração da SSR e dos Direitos à Saúde Sexual e Reprodutiva (DSSR)/VIH nos resultados da comparação das gravidezes não planeadas entre locais integrados e não integrados, vários estudos demonstraram uma redução nos custos da prestação de serviços, um aumento do conhecimento dos utentes e uma maior utilização de métodos contracetivos modernos (Haberlen, Narasimhan, Beres e Kennedy, 2017) [30]. Para alcançarem níveis mais elevados de eficiências operacionais, os executores do projeto podem aprender com os modelos de integração de serviços de sucesso documentados. Tal como reconhecido por Johnson, Varallyay e Ametep (2018), as recomendações políticas de governos, financiadores, organizações internacionais e organismos normativos apoiam explicitamente a integração da prestação de serviços de saúde reprodutiva e VIH, em particular de planeamento familiar. Reconhece-se que a integração é necessária para apoiar as necessidades de saúde reprodutiva das mulheres e dos homens, para fazer recuar a epidemia do VIH com o objetivo de pôr termo à SIDA como ameaça para a saúde pública e para chegar ao acesso universal à SSR até 2030, de acordo com os Objetivos de Desenvolvimento Sustentável (ODS) [1].

A compilação de artigos neste suplemento examina a integração da SSR e do VIH/SIDA de perspetivas diferentes. Da integração do planeamento familiar como segundo pilar da prevenção da transmissão vertical do VIH, nomeadamente entre trabalhadoras do sexo, ao conceito de gravidez mais segura em pessoas infetadas pelo VIH, passando pela integração da SSR no pacote mínimo da prevenção, cuidados e tratamento do VIH. Os artigos também examinam a conceção de abordagens inovadoras para a integração em modelos de prestação de serviços comunitários. A definição de sucesso na integração de serviços tem sido evasiva e as experiências partilhadas nestes artigos são transversais a vários países e focos geográficos, incluindo o Botsuana, Camarões, Quénia, Maláui, África do Sul, Tanzânia, Uganda e Estados Unidos. Além disso, uma revisão sistemática e uma revisão bibliográfica exploram, respetivamente, o problema específico da integração do teste do VIH no planeamento familiar e o problema da integração da SSR de adolescentes e mulheres jovens em cenários de emergência.

Debate

A integração eficaz dos serviços de VIH e SSR exige não só intervenções ao nível da mudança de comportamento dos prestadores de cuidados de saúde (Mudança de Comportamento dos Prestadores), como também uma maior compreensão dos beneficiários para uma resposta adequada às suas necessidades com base no seu conhecimento, atitude e perceção do risco. Tal como demonstrado por González, Kadengye e Mayega, (2019) no seu inquérito às famílias, representativo em termos nacionais, focado nos jovens ugandeses, os níveis elevados de conhecimento sobre SSR/VIH e de perceção do risco não travaram os

comportamentos sexuais de risco apesar do contexto de epidemia generalizada do VIH (prevalência de 2,1% entre jovens com 15-24 anos). Os autores afirmaram que as lacunas de eficácia na resposta integrada à SSR/VIH devem ser abordadas de uma forma global ao nível individual e estrutural [14].

Um grupo marginalizado entre populações-chave na África Subsariana, mulheres que consomem drogas injetáveis, mereceu atenção neste suplemento. Sylvia Ayon *et al.* (2019) recorreu à investigação de ação para identificar o processo, os impactos e os desafios da integração da SSR em programas comunitários de prevenção e redução dos danos do VIH nas cidades costeiras do Quénia. As conclusões desta investigação realçam a fraca utilização do planeamento familiar e de outros serviços de SSR, e dão uma visão crucial da aceitabilidade e das oportunidades para uma integração bem-sucedida ao nível da comunidade [25].

Um estudo transversal realizado no Quénia por Raymond Mutisya *et al.* avaliou o nível de integração do planeamento familiar em seis outras áreas de prestação de serviços (cuidados pré-natais, maternidades, cuidados pós-natais, assistência infantil, aconselhamento e despistagem do VIH, e serviços de VIH/SIDA em contextos de cuidados clínicos abrangentes). As conclusões do estudo confirmam o que já tinha sido documentado sobre a correlação positiva entre o conhecimento, as competências e a atitude, e a qualidade de serviço dos prestadores [21].

Uma revisão sistemática da integração dos serviços de despistagem do VIH nos serviços de planeamento familiar realizada por Narasimhan *et al.* (2019) revelou que o aconselhamento e a despistagem em matéria de VIH era tendencionalmente superior em locais integrados em comparação com locais não integrados, e as análises ajustadas através dos resultados variaram ligeiramente nos estudos. Analogamente às conclusões de Kiersten Johnson *et al.* esta revisão conclui que o progresso e sucesso globais na consecução dos objetivos em matéria de SSR e VIH depende dos progressos realizados na África Subsariana, onde as mulheres suportam o fardo pesado da gravidez indesejada e das infeções sexualmente transmissíveis, incluindo o VIH [22].

Num relatório de 2015 sobre o estado do mundo, o FNUAP afirmou que as inúmeras crises, guerras e desastres naturais em todo o mundo e, sobretudo, em África, estão a fazer com que as mulheres e as adolescentes enfrentem um risco significativamente acrescido de gravidez indesejada, morte materna, violência de género e infeção com VIH. Como tal, é pertinente que este suplemento inclua uma revisão bibliográfica realizada por Roxo, Walker, Mobula, Ficht e Yeiser, (2019) sobre a priorização da SSR e dos direitos das adolescentes e mulheres jovens nos serviços de tratamento e cuidados em cenários de emergência. Esta revisão revelou que o pluralismo de necessidades concorrentes em cenários de emergência remete para segundo plano o tempo e o espaço dedicados à integração eficaz das intervenções em matéria de VIH e SSR, e que é necessária uma maior vontade política para concretizar o programa de integração [26].

Dois dos artigos do suplemento debatem as principais conclusões sobre a disponibilidade dos serviços integrados relacionados com o VIH e planeamento familiar na África Subsariana, sendo que um dos artigos aborda a componente "qualidade". Estudos

realizados por Kanyangarara, Sakyi e Laar, (2019) e Barden-O'Fallon, Mejia e Close, (2019) restringiram a análise aos estabelecimentos de saúde que oferecem cuidados e assistência a doentes com VIH/SIDA, não só aconselhamento e despistagem em matéria de VIH, e serviços de PF. Recorrendo a uma análise secundária de Avaliações da Prestação de Serviços (SPA), Avaliações da Disponibilidade e Prontidão de Serviços (SARA) (2012 – 2015) de 10 países, incluindo seis na África Central e Ocidental), Kanyangara *et al*. descobriu que 93% dos estabelecimentos que oferecem serviços de cuidados e assistência a doentes com VIH também comunicaram a oferta de serviços integrados, mas apenas 29% foram classificados como tendo serviços de PF integrados no local, com base na disponibilidade das contribuições estruturais e do processo de prestação de cuidados (por exemplo, equipamento, diretrizes, prestadores de cuidados de saúde com formação e produtos de PF). Além disso, 94% dos estabelecimentos comunicaram a oferta regular de aconselhamento relativo a PF a utentes com VIH/SIDA e 80% tinham três ou mais métodos contracetivos em stock no momento dos inquéritos [23,29].

O alinhamento das medidas de SPA e uma investigação rápida dos indicadores de qualidade para a qualidade do PF em estabelecimentos de cuidados e assistência a doentes com VIH/SIDA de nível inferior integrados em comparação com não integrados no Maláui (2013 – 2014) e na Tanzânia (2014 – 2015), Close *et al*. revelou que 79% dos estabelecimentos no Maláui e 38% na Tanzânia oferecem serviços de PF. Em conformidade com o Quadro de cuidados de qualidade de Bruce/Jain, foram analisados 22 indicadores de qualidade e o estado de integração demonstrado foi substancialmente associado a três indicadores para o Maláui: "o estabelecimento tem todos os métodos (aprovados) disponíveis: sem roturas de stock", "o estabelecimento recebeu uma visita de inspeção nos últimos 6 meses", "observação e registo no processo clínico dos utentes". Para a Tanzânia, os indicadores associados ao estado de integração foram "o estabelecimento dispõe de armazenamento adequado de contracetivos e medicamentos", "o estabelecimento tem todos os métodos (aprovados) disponíveis: sem roturas de stock", "tempo de espera aceitável (negativo)" e "o estabelecimento tem mecanismos para a realização de alterações programáticas com base no feedback dos utentes" [23,29].

Dois dos artigos analisaram o conhecimento e o uso de métodos de conceção mais seguros entre os indivíduos infetados pelo VIH relativamente a como dar resposta às suas necessidades de conceção. Ambos os estudos realizados por Gwokyalya *et al*. (2019) e Schwartz *et al*. (2019) realçaram a necessidade de mais educação e da disponibilização de diferentes métodos de conceção mais seguros às pessoas que vivem com VIH. Das 5.198 mulheres inquiridas nos estabelecimentos de saúde do Uganda, 74,1 por cento tinham conhecimento de métodos de conceção mais seguros, mas o número diminui para 42 por cento nas mulheres com conhecimento de mais de um método. O estudo salientou igualmente a falta de envolvimento dos parceiros em casais serodiscordantes na escolha e intenção de uso de métodos de conceção mais seguros. Salientaram a falta de pessoal qualificado para se submeterem a alguns dos métodos de conceção mais seguros como, por exemplo, a lavagem de esperma. O estudo realçou os crescentes desafios enfrentados pelos indivíduos com VIH que pretendem ter filhos e concluiu que o conhecimento e uso de métodos de conceção mais seguros entre as mulheres seropositivas a receber cuidados de saúde é reduzido. Medidas destinadas a melhorar a divulgação do estatuto de portador do VIH, a integração da conceção mais segura nos serviços de PF e VIH, e esforços regionais no

sentido de promover a sensibilização e o acesso à conceção mais segura podem contribuir para aumentar a adoção de métodos de conceção mais seguros [24, 28].

Com a sua abordagem em quatro pilares, a PTV previne 90% de novas infeções pelo VIH entre as crianças, contribuindo, assim, para uma geração sem SIDA. A PTV está também na encruzilhada dos serviços de cuidados pré-natais e pós-natais, PF e serviços de prevenção, cuidados e tratamento do VIH. Três manuscritos neste suplemento abordam o problema da PTV na perspetiva da integração dos serviços de cuidados pré-natais, de VIH e de saúde sexual e reprodutiva. Os autores, Rwema *et al*. (2019), e Parmley *et al*. (2019), avaliaram a cadeia da PTV e investigaram os fatores que influenciam os serviços de cuidados pré-natais, na procura do comportamento num contexto de elevada prevalência do VIH entre as trabalhadoras do sexo em Port Elizabeth, na África do Sul [15, 16].

Rwema *et al*. (2019) descobriu que 61% das trabalhadoras do sexo estavam infetadas com o VIH e 52% delas sabiam do seu estatuto de portadoras do VIH antes do estudo. Foi encontrada uma lacuna de 40% em relação ao uso sistemático de preservativos pelas trabalhadoras do sexo não infetadas com o VIH com os seus clientes e uma lacuna de 43% no uso de métodos contracetivos modernos de longo prazo entre as trabalhadoras do sexo que vivem com VIH. Parmley *et al*. (2019) detetou, num contexto semelhante em Port Elizabeth, na África do Sul, uma descoberta tardia da gravidez (entre os 4 e 7 meses) entre as trabalhadoras do sexo que vivem com VIH e 40% delas recebem tratamento antirretroviral. Os fatores de influência identificados foram o abuso de álcool e de substâncias psicoativas, bem como a insatisfação com experiências de cuidados de saúde anteriores [15,16].

Dois manuscritos estão, respetivamente, relacionados com fatores associados à comunicação aos parceiros, no Botsuana, e à deteção precoce do VIH entre os filhos das trabalhadoras do sexo infetadas com o VIH, nos Camarões. Nos Camarões, das 481 trabalhadoras do sexo inquiridas no estudo elaborado por Rao *et al*., 70% comunicaram que nenhum dos seus filhos com idade inferior a 5 anos foi submetido ao teste do VIH. Os fatores que influenciaram o teste do VIH nos filhos das trabalhadoras do sexo seropositivas foram o recurso aos serviços pré-natais (OR ajustada de 2,12, IC de 95%: [1,02, 4,55]), saberem o seu estatuto de portadora do VIH (OR de 3,70 [2,30, 5,93]), a natureza desejada da gravidez (OR de 1,89 [1,16, 3,08]) e nível de educação superior (OR de 2,17 [1,01, 4,71]). A comunicação aos parceiros e o tratamento adequado dos parceiros são elementos essenciais para quebrar a cadeia de transmissão de infeções sexualmente transmissíveis (IST), incluindo a infeção pelo VIH. O suplemento inclui um estudo qualitativo realizado por A. Wynn et al. (2019), no Botsuana. O estudo revelou que o tratamento dos parceiros foi tardio e a maioria dos participantes manifestou uma preferência por comunicar a sua IST aos seus parceiros num estabelecimento de saúde com o apoio dos profissionais de saúde. Os autores concluíram que ainda há grandes progressos a fazer nos quatro pilares da PTV, sobretudo entre as populações-chave, como as trabalhadoras do sexo [17, 18].

O cancro do colo do útero é uma das principais causas de morte por cancro nas mulheres, sobretudo nos países de médio e baixo rendimento. Está associado a tipos persistentes ou de elevado risco (ou oncogénicos) do vírus do papiloma humano (HPV). A prevalência deste tipo de cancro é superior entre determinados grupos vulneráveis, como as pessoas que vivem com VIH. Este suplemento inclui um estudo original sobre a viabilidade, em zonas rurais do Zimbabué, da integração do rastreio do HPV nos serviços existentes de vacinação

no terreno e programas comunitários relacionados com o VIH. Foram recolhidas amostras a nível comunitário por trabalhadores comunitários de saúde com formação adequada. A recolha foi realizada durante visitas ao terreno agendadas para entrega de medicamentos antirretrovirais e vacinas infantis. Os trabalhadores comunitários de saúde explicaram como realizar a autorrecolha de amostras. Em seguida, as amostras foram transferidas para um estabelecimento de saúde para análise. Este modelo de alcance comunitário integrado foi aceite pelos beneficiários com uma taxa de participação de 82% [20].

Conclusão

Pôr fim à SIDA como ameaça para a saúde pública e garantir o acesso universal à saúde sexual e reprodutiva são as duas metas essenciais para o Objetivo de Desenvolvimento Sustentável (ODS) 3. Os artigos reforçam que, à medida que caminhamos para 2030, a integração dos DSSR e VIH pode desempenhar um papel importante na melhoria da saúde e do bem-estar de todos. Tal como Narasimhan *et al*. nos recordou, o progresso e sucesso globais na consecução dos objetivos em matéria de DSSR dependem dos progressos realizados na África Subsariana que tem a taxa mais elevada de gravidez indesejada e IST, incluindo o VIH.

Uma das principais mensagens que emerge do conjunto de artigos é a necessidade de aplicação do princípio da bidirecionalidade em todas as intervenções em matéria de DSSR e VIH. Estes artigos reforçam um dos pontos de ação-chave no *Apelo à ação para alcançar a cobertura universal dos serviços de saúde através de intervenções interligadas em matéria de DSSR e VIH,* lançado na Conferência Internacional sobre SIDA em 2018. Este apelo enfatiza que, para que as intervenções tenham o impacto desejado, precisam de envolver de forma significativa as comunidades na conceção, implementação e monitorização para fazer face às necessidades da comunidade.

Um traço comum é o facto de a integração apenas poder ser eficaz se forem feitos investimentos no desenvolvimento das capacidades dos profissionais de saúde e garantir que os estabelecimentos de saúde têm a infraestrutura necessária, estão bem equipados e dispõem de um stock adequado de produtos. É também evidente que os esforços de integração devem incluir não só os estabelecimentos de saúde, mas também intervenções de alcance comunitário.

Embora os ODS apelem à comunidade global para “não deixar ninguém para trás”, é necessário redobrar os esforços para dar resposta às necessidades das populações-chave e vulneráveis dentro dos quatro pilares da PTV e das adolescentes e mulheres jovens nos serviços de tratamento e cuidados em cenários de emergência. Este conjunto de artigos demonstra que reunir os DSSR e o VIH exigirá intervenções deliberadas e empenho político que coloque o indivíduo no centro da prestação de serviços.

Referências

1. Kiersten Johnson, Ilona Varallyay, Paul Ametepi. Integration of HIV and Family Planning Health Services in Sub-Saharan Africa: A Review of the Literature, Current Recommendations, and Evidence from the Service Provision Assessment Health Facility Surveys, ICF International, USA, 2018.

2. PRB, World Population Data Sheet, 2018.
3. United Nations, World Family Planning, 2017.
4. UNAIDS. Data 2018. Geneva: UNAIDS; 2018. http://www.unaids.org/sites/default/files/media_asset/unaids-data-2018_en.pdf.
5. The Glion Call to Action on Family Planning and HIV/AIDS in Women and Children 3-5 May 2004.
6. UNFPA 2016. Universal access to reproductive health. Progress and challenges. Geneva: UNFPA January 2016, p 16.
7. Hladik W, Stover J, Esiru G, Harper M, Tappero J (2009). The Contribution of Family Planning towards the Prevention of Vertical HIV Transmission in Uganda. PLoS ONE 4(11): e7691. doi:10.1371/journal.pone.0007691.
8. Vrazo AC, Sullivan D, Ryan Phelps B. Eliminating mother-to-child transmission of HIV by 2030: 5 strategies to ensure continued progress. Glob Health Sci Pract. 2018; 6 (2) : 249-256. https://doi.org/10.9745/GHSP-D-17-00097.
9. WHO. Preventing HIV and Unintended Pregnancies: Strategic Framework 2011–2015. Geneva: World Health Organization 2003, p85.
10. WHO. Strategic approaches to the prevention of HIV infection in infants: report of a WHO meeting, Morges, Switzerland, 20-22 March 2002. Geneva: World Health Organization 2003, p10.
11. Oladele Vincent Adeniyi, Anthony Idowu Ajayi, Mayowa Gabriel Moyaki, Daniel Ter Goon, Gordana Avramovic and John Lambert. High rate of unplanned pregnancy in the context of integrated family planning and HIV care services in South Africa. BMC Health Services Research (2018) 18:140. https://doi.org/10.1186/s12913-018-2942-z.
12. WHO. Preventing HIV and Unintended Pregnancies: Strategic Framework 2011–2015. Geneva: World Health Organization 2003.
13. Vrazo AC, Sullivan D, Ryan Phelps B. Eliminating mother-to-child transmission of HIV by 2030: 5 strategies to ensure continued progress. Glob Health Sci Pract. 2018; 6 (2) : 249-256. https://doi.org/10.9745/GHSP-D-17-00097.
14. P.R. González, D.T. Kadengye, R.W Mayega. The Knowledge-Risk-Behaviour Continuum among Young Ugandans: What it tells us about SRH/HIV Integration. BMC Public Health. 2019; 19 Suppl 1:S2
15. J. O. T. Rwema, S. Baral, S. Ketende, N. Phaswana-Mafuya, A. Lambert, Z. Khose, M. Mcingana, A. Rao, H. Hausler, S. Schwartz. Evaluating the Vertical HIV Transmission Risks and PMTCT cascades among South African Female Sex Workers. Have we forgotten PMTCT in their HIV programming? BMC Public Health. 2019; 19 Suppl 1:S3
16. L. Parmley, A. Rao, Z. Kose, A. Lambert, R. Max, N. Phaswanamafuya, M. Mcingana, H. Hausler, S. Baral, S. Schwartz. Antenatal care presentation and engagement in the context of sex work: Exploring barriers to care for sex worker mothers in South Africa. Reproductive Health. 2019; 16 Suppl 1:S4

17. A. Rao, S. Schwartz, S. C. Billong, A. Bowring, G. Fouda, F. Ndonko, I. Njindam, D. Levitt, A-C. Bissek, O. Njoya, S. Baral. Predictors of Early Childhood HIV Testing among Children of Sex Workers Living with HIV in Cameroon. BMC Public Health. 2019; 19 Suppl 1:S4
18. A. Wynn, C. Moucheraud, N. Moshashane, O. A. Offorjebe, D. Ramogola-Masire, J. D Klausner, C. Morroni. Using partner notification to address curable sexually transmitted infections in a high HIV prevalence context: A qualitative study about partner notification in Botswana. BMC Public Health. 2019; 19 Suppl 1:S5
19. N. Broutet, L. O'Neal Eckert, A. Ullrich, P. Bloem. Comprehensive cervical cancer control. A guide to essential practice. Geneva: World Health Organization 2014.
20. M. B. Fitzpatrick, Z. El-Khatib, D, Katzenstein, B. A. Pinsky, Z. M. Chirenje, K. McCarty. Community-Based Self-Collected Human Papillomavirus Screening in Rural Zimbabwe. BMC Public Health. 2019; 19 Suppl 1:S2
21. R. Mutisya, S. Karnad, J. Wambua, M. Kabue, F. Waweru, E. Omanga. Strengthening integration of family planning with HIV services: The Tupange project experience in three Kenyan cities. Reproductive Health. 2019; 16 Suppl 1:S6
22. M. Narasimhan, P. Teresa Yeh, S. Haberlen, C. E. Warren, C. E. Kennedy. Integration of HIV testing services (HTS) into family planning (FP) services: a systematic review. Reproductive Health. 2019; 16 Suppl 1:S5
23. J. Barden-O'Fallon, C. Mejia, M. A. Close. Quality of family planning services of HIV integrated and non-integrated facilities in Malawi and Tanzania. Reproductive Health. 2019; 16 Suppl 1:S3
24. S. Schwartz, N. Davies, N. Naidoo, D. Pillay, N. Sikhosana, S. Mullick. Clients' experiences utilizing a safer conception service for HIV affected individuals: Implications for differentiated care service delivery models. Reproductive Health. 2019; 16 Suppl 1:S9
25. S. Ayon, F. Jeneby, F. Hamid, A. Badhrus, T. Abdulrahman, G. Mburu. Developing integrated community-based HIV prevention, harm reduction, and sexual and reproductive health services for women who inject drugs. Reproductive Health. 2019; 16 Suppl 1:S2
26. U. Roxo, D. Walker, Linda Mobula, Allison Ficht, Sarah Yeiser. Prioritizing the Sexual Reproductive Health and Rights of Adolescent Girls and Young Women within HIV Treatment and Care Services in Emergency Settings: A Girl-Centered Agenda. Reproductive Health. 2019; 16 Suppl 1:S1
27. V. Gwokyalya, J. Beyeza-Kashesya, J. B. Bwanika, J. KB Matovu, S. Mugerwa, J. Arinaitwe, D. Kasozi, J. Bukenya, R. Kindyomunda, G J. Wagner, F E. Makumbi and R K. Wanyenze. Knowledge and correlates of use of safer conception methods among HIV-infected women attending HIV care in Uganda. Reproductive Health. 2019; 16 Suppl 1:S8
28. M. Kanyangarara, K. Sakyi, A. Laar. Availability of integrated family planning services in HIV care and support sites in sub-Saharan Africa: a secondary analysis of national health facility surveys. Reproductive Health. 2019; 16 Suppl 1:S4

29. Sabina A. Haberlen, Manjulaa Narasimhan, Laura K. Beres, and Caitlin E. Kennedy. Integration of Family Planning Services into HIV Care and Treatment Services: A Systematic Review. Studies in Family Planning, 2017.

Sobre este suplemento

Este artigo foi publicado como parte da revista científica *BMC Public Health*, Volume 19 Suplemento 1, 2019: Integração Eficaz dos Serviços de Saúde Sexual e Reprodutiva e de Prevenção, Cuidados e Tratamento do VIH na África Subsariana: Onde estão as provas da implementação do programa?
O suplemento foi publicado como uma colaboração entre as revistas científicas *Reproductive Health* e *BMC Public Health.* O conteúdo integral, incluindo as versões em francês, português e inglês, estão disponíveis online aqui:
https://bmcpublichealth.biomedcentral.com/articles/supplements/volume-19-supplement-1
e
https://reproductive-health-journal.biomedcentral.com/articles/supplements/volume-16-supplement-1

Declarações

Aprovação da Comissão de Ética e consentimento para participação
Não aplicável

Consentimento para publicação
Não aplicável

Disponibilidade dos dados e materiais
Não aplicável

Interesses concorrenciais
Os autores declaram que não têm interesses concorrenciais.

Financiamento
O suplemento da revista científica foi possibilitado pelo apoio generoso do povo americano através da Agência dos Estados Unidos para o Desenvolvimento Internacional (USAID) em parceria com o Fundo das Nações Unidas para a População (FNUAP) e o Programa Conjunto das Nações Unidas sobre VIH/SIDA (ONUSIDA).
Os pontos de vista expressos nesta publicação são meramente as opiniões dos autores e não refletem necessariamente as políticas oficiais da USAID, do FNUAP ou da ONUSIDA, nem a referência a nomes de departamentos ou agências implica o apoio do Governo dos EUA, do FNUAP ou da ONUSIDA.

Contribuições dos autores

DMK contribuiu para a introdução do editorial e o debate sobre os manuscritos relacionados com a integração dos serviços de SSR e VIH em geral. HG contribuiu para a introdução e o debate sobre os manuscritos relacionados com as trabalhadoras do sexo e a integração dos serviços de cuidados pré-natais/VIH. SM contribuiu para o debate sobre os manuscritos relacionados com a disponibilidade e qualidade da integração dos serviços de SSR e VIH. AB contribuiu para o debate sobre os manuscritos relacionados com a conceção segura e as pessoas que vivem com VIH. RD contribuiu para a secção de conclusão. Todos os autores leram e aprovaram o editorial final.

Agradecimentos
O comité editorial do Suplemento gostaria de expressar o seu profundo agradecimento a todos os colegas avaliadores pela sua generosidade em termos de tempo e conhecimento. O comité também gostaria de agradecer os contributos essenciais de Susan Mathew, Jennifer Mason, Nithya Mani e Sheryl Martin durante as diferentes fases do processo de avaliação pelos pares e a edição final deste editorial.

Os pontos de vista expressos nesta publicação são meramente as opiniões dos autores e não refletem necessariamente as políticas oficiais da USAID, do FNUAP ou da ONUSIDA, nem a referência a nomes de departamentos ou agências implica o apoio do Governo dos EUA, do FNUAP ou da ONUSIDA.

Informação sobre os autores

O Dr. Didier Mbayi Kangudie, MD, MPH (mkangudie@usaid.gov) é Conselheiro Principal para a Saúde, USAID/África Ocidental, Gabinete Regional da Saúde. O Dr. Hugues Guidigbi, MD, MPH (hguidigbi@usaid.gov) é Conselheiro Principal em matéria de VIH/SIDA, USAID/África Ocidental, Gabinete Regional da Saúde. Sheila Mensah (smensah@usaid.gov) é Conselheira Principal de Comunicações, Monitorização e Avaliação, USAID/África Ocidental, Gabinete Regional da Saúde. Abdul A. Bala (abala@usaid.gov) é Analista de Programas e Investigação, USAID/África Ocidental, Gabinete Regional da Saúde. Richard Delate (delate@unfpa.org) é Especialista do Programa SSR/VIH, FNUAP, Gabinete Regional da África Oriental e Austral
